# An Investigation of Modifying Effects of Metallothionein Single-Nucleotide Polymorphisms on the Association between Mercury Exposure and Biomarker Levels

**DOI:** 10.1289/ehp.1104079

**Published:** 2012-01-09

**Authors:** Yi Wang, Jaclyn M. Goodrich, Brenda Gillespie, Robert Werner, Niladri Basu, Alfred Franzblau

**Affiliations:** 1 Department of Environmental Health Sciences,; 2 Department of Biostatistics, and; 3 Department of Physical Medicine and Rehabilitation, University of Michigan, Ann Arbor, Michigan, USA

**Keywords:** biomarker, gene–environment interaction, mercury, metallothionein, single-nucleotide polymorphism

## Abstract

Background: Recent studies have suggested that several genes that mediate mercury metabolism are polymorphic in humans.

Objective: We hypothesized that single-nucleotide polymorphisms (SNPs) in metallothionein (MT) genes may underlie interindividual differences in mercury biomarker levels. We studied the potential modifying effects of MT SNPs on mercury exposure–biomarker relationships.

Methods: We measured total mercury in urine and hair samples of 515 dental professionals. We also surveyed occupational and personal exposures to dental amalgam and dietary fish consumption, from which daily methylmercury (MeHg) intake was estimated. Log-transformed urine and hair levels were modeled in multivariable linear regression separately against respective exposure surrogates, and the effect modification of 13 MT SNPs on exposure was investigated.

Results: The mean mercury levels in urine (1.06 μg/L) and hair (0.51 μg/g) were not significantly different from the U.S. general population (0.95 μg/L and 0.47 μg/g, respectively). The mean estimated daily MeHg intake was 0.084 μg/kg/day (range, 0–0.98 μg/kg/day), with 25% of study population intakes exceeding the current U.S. Environmental Protection Agency reference dose of 0.1 μg/kg/day. Multivariate regression analysis showed that subjects with the *MT1M* (rs2270837) AA genotype (*n* = 10) or the *MT2A* (rs10636) CC genotype (*n* = 42) had lower urinary mercury levels than did those with the *MT1M* or *MT2A* GG genotype (*n* = 329 and 251, respectively) after controlling for exposure and potential confounders. After controlling for MeHg intake, subjects with *MT1A* (rs8052394) GA and GG genotypes (*n* = 24) or the *MT1M* (rs9936741) TT genotype (*n* = 459) had lower hair mercury levels than did subjects with *MT1A* AA (*n* = 113) or *MT1M* TC and CC genotypes (*n* = 15), respectively.

Conclusion: Our findings suggest that some MT genetic polymorphisms may influence mercury biomarker concentrations at levels of exposure relevant to the general population.

Large interindividual variation has been observed in urinary mercury levels in the general population and in workers after exposures of similar magnitudes to elemental mercury (Tsuji et al. 2003), and in hair mercury levels in association with dietary fish consumption (Canuel et al. 2006; Haxton et al. 1979). Variation was also seen in the elimination half-life of methylmercury (MeHg) in humans, ranging from 45 to 70 days (Clarkson 2002).

Although variation in sources and levels of exposure may contribute to the overall interindividual variation in mercury biomarker levels, differences in mercury retention may also play an important role. Mercury retention may be influenced by changes in mercury binding by functional enzymes and proteins that transport, oxidize, and reduce mercury and its metabolites in humans (Gundacker et al. 2010). Single-nucleotide polymorphisms (SNPs) in genes that encode the enzyme for the rate-limiting step in glutathione synthesis (glutamate cysteine ligase) and that catalyze glutathione conjugation [glutathione *S*-transferase (GST)] are associated with variability in mercury biomarker levels after exposures to MeHg or inorganic mercury (Custodio et al. 2004, 2005; Gundacker et al. 2007, 2009; Schläwicke Engström et al. 2008).

Little is known about how polymorphisms in genes encoding metallothioneins (MTs), a family of thiol-rich mercury-binding proteins, may affect mercury biomarker levels in humans. MT proteins actively bind heavy metals via thiol groups in cysteine residues and protect against heavy metal toxicity and oxidative stress in kidney, liver, and brain (Aschner et al. 2006; Kumari et al. 1998; Schurz et al. 2000; Yoshida et al. 1999). Humans express four primary MT isoforms (MT1, MT2, brain-specific MT3, and MT4). MT transcription levels could affect their mercury-binding capacity. SNPs located in regions important for regulating transcription may have an impact on MT detoxifying capability, subsequently affecting mercury retention and altering biomarker levels.

Few studies have investigated the potential effect modification of MT SNPs on the relationship of urinary mercury levels with elemental mercury exposures. Gundacker et al. (2009) investigated the effects of MT SNPs on the association of MeHg exposure with hair mercury levels and found that subjects with the *MT4* [rs11643815, dbSNP database (National Center for Biotechnology Information 2011)] GA or AA variant genotype had lower hair mercury levels. The goal of the present study was to investigate whether SNPs in MT and MT transcription factor genes modify the relationships of elemental mercury and MeHg exposure with urinary and hair mercury levels, respectively. We sought to explain the considerable variation in biomarkers seen in subjects exposed to elemental mercury and MeHg of similar magnitudes by studying polymorphisms in genes that play key roles in mercury toxicokinetics.

## Materials and Methods

Subjects were recruited during the Michigan Dental Association annual conventions held in 2009 (*n* = 232) and 2010 (*n* = 283), as previously described (Goodrich et al. 2011). They represent a convenience sample of dental professionals attending the conventions. All participants provided written informed consent. The study was approved by the University of Michigan Institutional Review Board.

*Measurements of exposure.* Each subject completed a self-administered questionnaire to provide information about recent mercury exposures from different sources, demographic information, and covariates. Subjects reported elemental mercury exposures as average number of amalgams placed or removed per week (amalgams handled) and total number of dental amalgam restorations in their mouth (personal amalgam). We also surveyed MeHg exposure from dietary fish consumption within the 6-month period before the date of the survey. We surveyed the average portion size and consumption frequency of 28 fish species [see Supplemental Material, Table 1 (http://dx.doi.org/10.1289/ehp.1104079)], using a scheme adopted from the National Health and Nutrition Examination Survey (NHANES) Food Frequency Questionnaire (2003–2004) [Centers for Disease Control and Prevention (CDC) 2003]. We also obtained species-specific average mercury concentrations (see Supplemental Material, Table 1). We estimated daily MeHg intake (micrograms per kilogram per day) from dietary fish consumption for each subject based on the formula


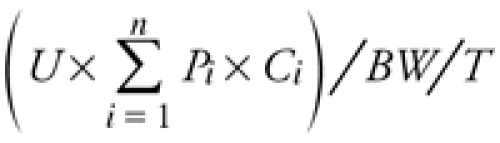
[1]

**Table 1:** Selected MT SNPs genotyped in 2009 and 2010.

3´ UTR					Missense				MRE proximity (5´ near)	
*MT2A*	*MT1M*	*MT1M*	*MT1G*	*MT1E*	*MT1A*	*MT1M*	*MT4*	*MT1A*	*MT2A*	*MT1A*	*MTF1*	
dbSNP no.		rs10636	rs9936741	rs2270837	rs12315	rs708274		rs11640851	rs1827210	rs11643815	rs8052394		rs28366003	rs9922957		rs473279	rs3748682
Major allele		G	T	G	G	G		A	A	G	A		A	C		G	T
Allele variant		C	C	A	T	T		C	C	A	G		G	G		A	C
Minor allele frequency		0.299	0.020	0.153	0.048	0.126		0.344	0.157	0.136	0.095		0.062	0.127		0.334	0.244
Genotype year																	
2009 only					X	X			X	X							X
2010 only											X		X	X			
2009–2010		X	X	X				X								X	


where *U* is the average unit portion size of fish meals (grams per portion); *P_i_* is the frequency of eating a particular fish species (portions per month), with *i* = 1, 2, 3 . . . 28 species; *C_i_* is the species-specific average MeHg concentration in fish tissues (micrograms per gram); *BW* is body weight of the subject (kilograms); and *T* is 30 days/month.

*Other covariates.* We classified participant job categories as dentist, hygienist, dental assistant, and other. Marketing managers or exhibitors, who do not have direct contact with mercury but are affiliated with a dental office or an organization, were categorized as “other.” We also obtained other covariates, including alcoholic beverage consumption, teeth grinding while sleeping, gum chewing (hours per day), and past chelation therapy.

*Urine and hair specimens.* Each subject provided a spot urine sample in a mercury-free container (Vacutainer Urine Collection Cup; Becton Dickinson, Franklin Lakes, NJ). A minimum of 10 mg of hair (~ 10–20 hair strands) was collected from the occipital region of the head. We were not able to obtain urine from 13 subjects and hair samples from 10 subjects.

Total mercury content in urine and hair (first 2 cm of hair closest to the scalp) samples was determined using atomic absorption spectroscopy (Direct Mercury Analyzer-80; Milestone Inc., Shelton, CT) based on U.S. Environmental Protection Agency (EPA) Method 7473, as described elsewhere (Goodrich et al. 2011). No hair or urine samples were below the theoretical method detection limit (urine: 2009, 0.05 ng; 2010, 0.01 ng; hair: 2009, 0.07 ng; 2010, 0.01 ng), calculated as three times the standard deviation of blank measurements. We measured specific gravity of urine samples to account for the variability of metal excretion associated with spot urine samples (Mason and Calder 1994). Creatinine was not measured (Heyer et al. 2007).

*SNP selection and genotyping.* Buccal swabs were used to collect DNA samples (Goodrich et al. 2011). Genomic DNA was isolated and purified for genotyping using the Wizard SV Genomic DNA Purification System (Promega, Madison, WI).

Thirteen MT SNPs were selected in regions that were important for gene expression or were hypothesized to regulate the structure and/or folding of the MT proteins (e.g., exon coding regions) (Table 1). SNPs in these regions might lead to alterations in mercury-binding capacity, subsequently influencing biomarker levels. Thus, we included missense SNPs with relatively high prevalence in the coding regions and SNPs located in regions important for mRNA transcription, including the 5´ flanking region of the sequences (Aschner 1996), the metal-responsive elements (MREs) in the upstream promoter region (Karin et al. 1987a), and the 3´ untranslated region (3´ UTR) (Hesketh 2004). The availability and use of transcription inducers, such as metal-regulatory transcription factor-1 (MTF-1), are important for MT expression, and SNPs in *MTF1* were also genotyped (Karin et al. 1987b; Palmiter 1994). All selected SNPs had minor allele frequencies ≥ 5% in the Centre d’Etude du Polymorphisme Humain panel (Dausset et al. 1990), and all were in Hardy-Weinberg equilibrium. Not all SNPs were genotyped in subjects from both sampling years (Table 1).

We used TaqMan allelic discrimination assays (Applied Biosystems, Foster City, CA) to genotype all SNPs except *MT1A* (rs8052394), and results were read on a 7300 Real-time PCR (polymerase chain reaction) System (Life Technologies Corp., Carlsbad, CA). The restriction fragment length polymorphism (RFLP) method was used to genotype *MT1A* (rs8052394).

*Statistical methods.* All statistical analyses were performed using SAS software (version 9.2; SAS Institute Inc., Cary, NC). In all analyses, we excluded four subjects who reported chelation therapy in the last 6 months. Eight subjects who reported a history of preexisting kidney disease (lithiasis, pyelonephritis, orthostatic proteinuria, end-stage kidney disease, or chronic renal failure) were excluded from all analyses of urine mercury. In our sample, dentists had a higher mean urine mercury level (1.40 μg/L) than did hygienists (0.64 μg/L) or assistants (0.96 μg/L). To investigate the hypothesis and ensure coherence in discussion, we investigated MT SNP effect modification in dentists, the subpopulation with the highest elemental mercury exposure, assuming the same toxicokinetics in dentists, hygienists, and assistants. A dichotomous variable with dentist being the referent, as opposed to nondentist (hygienist, assistant, and other), was thus used in the analyses.

Descriptive analyses were performed on body mass index (BMI), age, occupation, and race (Caucasian and non-Caucasian) along with mercury exposure levels reflected in urinary and hair mercury biomarkers and exposure surrogate variables (e.g., amalgams, fish consumption). Bivariate analyses included race- and occupation-stratified analyses of BMI and age, exposure-stratified urinary and hair mercury levels, and SNP-genotype–stratified urinary and hair mercury levels. Multivariate regression analyses were conducted in two phases for log-transformed urine and hair mercury levels because both urinary and hair mercury levels were not normally distributed. We fitted urine regression models with unadjusted mercury levels and with those adjusted for specific gravity (1.017). Parameter estimates did not notably change, and corresponding significance changed in only a few instances in specific-gravity–adjusted models. Unadjusted models are reported here unless otherwise noted (Heyer et al. 2007).

In the first phase, using multivariate linear regression models, natural log (ln)-transformed urinary and hair mercury were regressed separately against number of personal amalgams (linear continuous) and number of amalgams handled per week [ordinal; see categories in Supplemental Material, Table 2 (http://dx.doi.org/10.1289/ehp.1104079)] and estimated daily MeHg intake from fish (linear continuous). In the regression models, the covariates gum chewing, teeth grinding, occupation, age, race, BMI, sex, and alcohol consumption were all added to both urine and hair models. A base model was selected separately for each biomarker using backward elimination starting from the model that included all exposure terms and covariates. The least statistically significant predictor was eliminated in each step, and the final base models for urine and hair mercury were derived by retaining all significant predictors (*p* < 0.05). Sex was forced into both final models to assess the potential confounding of sex with occupation because most of the nondentists (94.8%) were female. We fitted each base model with a dentist-only or a nondentist-only sample; sex was not significant in the dentist-only sample, meaning the significance of sex was due to confounding with occupation. Thus, we excluded sex in the final base models. In the second phase, we created two dummy variables for each SNP: heterozygote (major homozygote as referent) and homozygote variant (major homozygote as referent). The base models of urinary and hair mercury were combined with main effect and interaction terms between the respective exposure predictors and dummy variables of each SNP. Interactions between exposure and genotype were investigated one SNP at a time.

**Table 2:** Demographics.

Characteristic	*n*	Age [years (mean ± SD)]	BMI [kg/m^2^ (mean ± SD)]	Female [*n* (%)]
Occupation								
Dentist		244		56.1 ± 11.6*		26.4 ± 4.0		60 (24.6)
Nondentist		269		48.2 ± 11.2*		26.4 ± 5.3		255 (94.8)
Subtotal		513						315 (61.4)
Missing		2						
Race								
Caucasian		463		52.5 ± 11.9**		26.3 ± 4.6		
Non-Caucasian		49		46.8 ± 12.5**		27.1 ± 6.1		
Subtotal		512						
Missing		3						
*p < 0.005. **p < 0.0001.								

## Results

*Demographics, exposure surrogates, and biomarkers.* The total sample included 515 participants and was predominantly Caucasian (90.5%). Dentists comprised 47.4% of the sample, and most of the nondentists were female (Table 2). Mean age differed significantly within both race and occupation categories. The mean numbers of amalgams handled per week and personal amalgams were 25.5 and 4.1, respectively. The mean estimated daily MeHg intake from fish was 0.084 μg/kg/day (range, 0–0.98 μg/kg/day), with 25% of study population intakes exceeding the current U.S. EPA reference dose of 0.1 μg/kg/day (U.S. EPA 2001).

The mean levels and distribution of urine and hair mercury seen in our study were similar to those in the U.S. general population (Table 3) because no significant difference was found in geometric and arithmetic mean mercury levels in urine or hair in our study population compared with reference levels reported for NHANES 2003–2004 (CDC 2009) and NHANES 1999–2000 (McDowell et al. 2004), respectively. Mean urinary mercury levels showed a linear trend of increase as exposure increased from occupational handling of amalgam and personal amalgam [see Supplemental Material, Table 2 (http://dx.doi.org/10.1289/ehp.1104079)]. Similarly, hair mercury levels increased linearly with MeHg intake from dietary fish consumption (see Supplemental Material, Table 3).

**Table 3:** Urine mercury (μg/L) and hair mercury (μg/g) in the Michigan Dental Association (MDA) mercury study compared with reference levels from NHANES (2003–2004) and NHANES (1999–2000), respectively.

Geometric mean	Arithmetic mean*a*	Percentile						
		Biomarker, study	50th	75th	90th	95th
Urine mercury												
NHANES 2003–2004 (*n* = 1,529)		0.50^#^		0.95		0.48		1.12		2.20		3.33
MDA study												
2009 (*n* = 229)		0.69^##^		1.11		0.72		1.37		2.51		3.37
2010 (*n* = 273)		0.62^##^		1.02		0.62		1.19		2.15		3.74
2009–2010 (*n* = 502)		0.65^#^		1.06		0.66		1.29		2.34		3.37
Hair mercury												
NHANES 1999–2000 (*n* = 1,726)		0.12*		0.47		0.19		0.42		1.11		1.73
MDA study												
2009 (*n* = 226)		0.30**		0.55		0.29		0.66		1.36		1.92
2010 (*n* = 279)		0.27**		0.45		0.28		0.54		1.07		1.33
2009–2010 (*n* = 505)		0.28*		0.51		0.29		0.58		1.17		1.49
*a*Urine arithmetic mean was calculated using NHANES (2003–2004) data. *p = 0.29. **p = 0.90. ^#^*p* = 0.19. ^##^*p* = 0.77.												

*Associations between SNPs and biomarker levels with and without adjustment for exposure.* Mean urine and hair mercury levels were compared among SNP genotypes with no adjustment for amalgam exposure or dietary MeHg intake, respectively. We observed no significant differences for any genotype [see Supplemental Material, Table 4 (http://dx.doi.org/10.1289/ehp.1104079)].

**Table 4:** Coefficients and *p*-values of multivariate linear regression models of ln-transformed urinary mercury predicted against exposure surrogates of elemental mercury, *MT1M* [3´ UTR (G > A); rs2270837] genotype, and exposure–*MT1M* interactions.

Exposure	β-Coefficient	*p*-Value
Base model (*R*^2^ = 0.25)				
Intercept		–0.70		
Personal amalgams		0.085		< 0.0001
Amalgams handled/week		0.11		0.04
Nondentist		–0.38		0.001
SNP main effects				
Heterozygote		–0.04		0.87
Homozygote variant		1.85		0.008
SNP–exposure interaction				
Personal amalgams × heterozygote		–0.002		0.92
Amalgams handled × heterozygote		0.11		0.32
Nondentist × heterozygote		–0.03		0.90
Personal amalgams × homozygote variant		–0.25		0.02
Amalgams handled × homozygote variant		–0.97		0.01
Nondentist × homozygote variant		–0.06		0.94


In the base urine model, number of personal amalgams and number of amalgams handled per week predict ln-transformed urinary mercury with adjustment for occupation. Occupational mercury exposure explains only approximately 10% of the total variance of the multivariate model, compared with > 60% for exposure from personal amalgams. We added SNP main effect and interaction terms to each base model. Statistically significant interactions were observed for *MT1M* (rs2270837) (Table 4) and *MT2A* (rs10636), although the latter (homozygote variant CC interacting with personal amalgam exposure; β = 0.06) was observed only when urine was adjusted for specific gravity. Compared with the *MT1M* (rs2270837) homozygote genotype GG, subjects with homozygote variant genotype AA had lower urinary mercury levels [Table 4; see Supplemental Material, Figure 1a,b (http://dx.doi.org/10.1289/ehp.1104079)].

In the hair mercury base model, estimated daily MeHg intake from fish predicted ln-transformed hair mercury after adjusting for occupation and age. In the subsequent analyses, only estimated daily MeHg intake was used as a predictor because we sought to investigate the effect modification of SNPs on estimated daily MeHg intake and to simplify interpretation. We then added SNP main effect and interaction terms to each base model. For all SNPs except *MT1A* (rs8052394) and *MT1M* (rs9936741), there were no significant interaction terms. Compared with homozygote *MT1M* (rs9936741; TT), after controlling for MeHg intake, subjects with heterozygote TC genotype had higher hair mercury levels [Table 5; see Supplemental Material, Figure 1c (http://dx.doi.org/10.1289/ehp.1104079)]. Those with *MT1A* (rs8052394) heterozygote GA and homozygote variant GG genotypes had lower hair mercury levels than did those with homozygote AA genotype (Table 5; see Supplemental Material, Figure 1d).

**Table 5:** Coefficients and *p*-values from multivariate linear regression models of ln-transformed hair mercury predicted by estimated MeHg exposure, SNP genotype, and intake–SNP interactions.

*MT1M* 3´ UTR (T > C; rs9936741; *R*^2^ = 0.20)			*MT1A* missense (A > G; rs8052394; *R*^2^ = 0.21)		
β-Coefficient	*p*-Value	β-Coefficient	*p*-Value
Base model								
Estimated daily MeHg intake		3.69		< 0.0001		4.04		< 0.0001
SNP main effects								
Heterozygote		–0.03		0.92		0.21		0.40
Homozygote variant		*—a*		—*a*		—*b*		—*b*
SNP–intake interactions								
Intake × heterozygote		19.3		0.02		–300		0.02
Intake × homozygote variant		—*a*		—*a*		—*b*		—*b*
**a**The homozygote variant number was zero for *MT1M* (rs9936741). **b**Because the number of homozygote variants for *MT1A* (rs8052395) was too small (*n* = 1), it was lumped with the heterozygote variant in testing interaction.								

## Discussion

We found significant effect modification of *MT1M* (rs2270837) homozygote variant AA genotype on the relationship of urinary mercury level with both occupational and personal exposures to elemental mercury after adjusting for covariates. We also found significant effect modification of *MT2A* (rs10636) CC genotype on the relationship between urinary mercury level and personal exposure to elemental mercury. We found significant effect modification of *MT1M* (rs9936471) heterozygous TC genotype, and *MT1A* (rs8052394) pooled heterozygote GA and homozygote variant GG genotypes on the relationship of hair mercury level with estimated daily MeHg intake from fish consumption. No significant effect modification was found from other MT SNPs.

The mean urinary mercury levels observed in our study population (dentists, 1.37 μg/L; hygienists and assistants, 0.75 μg/L) were lower than those in some previous reports for both occupationally and nonoccupationally exposed populations (dentists, 2.50–3.32 μg/L; dental assistants, 1.60–1.98 μg/L) (DeRouen et al. 2006; Echeverria et al. 2005,^2006^; Factor-Litvak et al. 2003; Heyer et al. 2008). The relatively low hair mercury levels and estimated mean daily MeHg intake from fish were consistent with what has been reported in North American nonindigenous populations in a number of studies (0.068 ± 0.109 μg/kg/day) (Canuel et al. 2006; Mahaffey et al. 2004). In our study, where occupation was a predictor of hair mercury level, it may be that occupation was a surrogate of socioeconomic status (SES), because dentists fall into a higher SES group and were more likely to eat fish than were nondentists. Increasing age has been shown to be associated with increasing MeHg level in blood (Schläwicke Engström et al. 2008) and may also be a reflection of deterioration of mercury metabolism and elimination.

Prior epidemiological literature on the studied MT SNPs and their relationship with mercury exposure is scarce. Gundacker et al. (2009) reported findings of effect modifications of *MT4* (rs11643815) on the exposure–biomarker relationship for mercury in hair but not in urine. Unlike Gundacker et al. (2009), we did not assume that all fish have the same level of mercury. We did not find any effect modification of *MT4*, which differs from the finding of Gundacker et al. (2009). This may be the result of using species-specific fish mercury levels in our calculation of estimated daily MeHg intake, which is likely a better estimation than total fish meals used in their study.

Here we studied several other MT SNPs hypothesized to be potentially important for their relationship with mercury exposure, some of which showed significant interactions with the exposure–biomarker relationship. We found the exon-coding *MT1A* (rs8052394) to modify the relationship of MeHg intake with hair mercury. *MT1A* isoforms are functional (West et al. 1990), but knowledge of the impact of the various SNPs, including *MT1A* (rs8052394), on structural/folding changes and the resulting impact on protein functionality and ability to bind mercury is limited.

Differential findings of the effects of the SNPs on elemental-mercury–biomarker (urine) and MeHg–biomarker (hair) relationships are not surprising given that the binding of heavy metals varies depending on the molecular structures and redox chemistry of MT proteins (Krezel and Maret 2007). Notably, we did not observe the positive effect modification of *MT1M* (rs2270837) on urine mercury in the hair mercury model, and vice versa for *MT1M* (rs9936741). Prior evidence of *MT1M* SNP functionality is lacking because the MT1M isoform has only recently been found to be functional (Lin et al. 2009; Michael et al. 2011; Oliveira et al. 2011). Hence, the significance of these associations is uncertain. Although other isoforms, including MT2A, MT1E, and MT1G, are also functional (West et al. 1990), their ability to bind heavy metals, as with the MT1A isoform, depends on several factors: MT abundance in target tissues, mercury form in target tissues, and redox chemistry of MT and thiolate (Maret and Vallee 1998). Inorganic mercury and MeHg differ in their target organs (kidney vs. brain) and elimination routes (urine vs. feces). Thus, levels of MT vary across the target tissues, which may contribute to the differential modifications. Overall, the underlying mechanism(s) for the observed significant interactions with exposure–biomarker relationship is unclear and requires further study.

The present study has a number of limitations. First, the study has a relatively small sample size. For some SNPs [e.g., *MT1M* (rs2270837)], < 15 subjects had the homozygous variant genotype. The small numbers limit the power to assess effects of gene–gene interactions on exposure–biomarker relationships. Second, it was difficult to rule out the possibility of false positives due to multiple comparisons. Of the 51 comparisons made in the urine models, only 2 were statistically significant [see Supplemental Material, Table 5 (http://dx.doi.org/10.1289/ehp.1104079)]; 2 of 19 comparisons made in the hair models were significant (see Supplemental Material, Table 6). Third, our study group was a convenience sample, not a random sample. However, there is no reason to believe that subjects had any prior knowledge of their genotypes or mercury levels. Thus, there was a low probability of selection bias. Fourth, instead of using total amalgam surfaces, a more refined and potentially more accurate surrogate for personal exposure to elemental mercury, we used the total number of amalgam restorations. Despite this, the total number of personal amalgams was still found to be the most significant predictor for ln-transformed urinary mercury levels. Fifth, the choice of deriving daily MeHg intake from the NHANES Food Frequency Questionnaire (2003–2004; CDC 2004) and species-specific mercury levels may create recall and misclassification biases. Despite similar bias in self-reporting recent exposure to elemental mercury, our findings on the relationship between urinary mercury levels and exposures from personal amalgams and dental practice were consistent with the results of previous reports. Sixth, mercury measurements in 2 cm of hair closest to the scalp reflect only the most recent 2 months of MeHg exposure from fish. The questionnaire examined fish consumption during the 6 months before the survey, and an unbiased reflection of fish MeHg intake in hair mercury was dependent upon a steady-state body burden of MeHg. The fish consumption of subjects may have fluctuated in the months prior to the survey. However, such fluctuation would likely bias the study results toward the null because a subject’s fish consumption may either increase or decrease during the 2–6 months before the survey. Seventh, day-to-day variability in urinary mercury excretion has been reported to average 22% among samples taken on three consecutive days (Ellingsen et al. 1993). However, this magnitude of variation is modest, and the likely impact would be to bias the results toward the null. Despite the study limitations, this is the largest gene–environment study investigating the potential impact of MT SNPs in humans on the relationship between mercury biomarkers and exposure to both elemental mercury and MeHg.

## Conclusion

We observed significant effect modifications of MT SNPs on biomarker–exposure associations for both elemental mercury and MeHg. Our study is the first to report significant effect modification of selected MT SNPs on the relationship of urinary mercury with occupational and nonoccupational exposures. We used a more precise survey methodology for estimating individual daily MeHg intake from fish based on the NHANES Food Frequency Questionnaire (2003–2004; CDC 2004) and species-specific mercury levels. The effect modifications of some MT SNPs appear to differ on the basis of mercury forms, although the exact mechanism is unclear. Thus, our findings contribute to a small body of previous research on MT SNPs involved in modifying the mercury exposure–biomarker relationship in humans, and also form a basis for future work in the field of gene–environment interactions concerning mercury. The ultimate goal is to improve our understanding of mercury biomarkers and the overall risk assessment of mercury exposure. Future studies are warranted to replicate the effect modification results of the SNPs and to assess the potential mechanisms underlying the effect modifications (e.g., altered expression). Future work may also examine gene–gene interactions (e.g., GST) with MT SNPs on the exposure–biomarker (e.g., blood) relationship in a larger sample. Although this study focused on dental professionals, the findings are relevant to the U.S. general population.

## Supplemental Material

(221 KB) PDF
