# Erratum

**DOI:** 10.1289/ehp.121-a13

**Published:** 2013-01-01

Wang et al. have reported an error in their article, “An Investigation of Modifying Effects of Metallothionein Single-Nucleotide Polymorphisms on the Association between Mercury Exposure and Biomarker Levels” [Environ Health Perspect 120:530–534 (2012)]. Throughout the article, the SNP labeled “rs2270836” should have been “rs2270837.” This does not alter any conclusions of their paper.

The authors regret the error.

