# Supplementary material for: An Investigation of Modifying Effects of Metallothionein Single-Nucleotide Polymorphisms on the Association between Mercury Exposure and Biomarker Levels
Source: Environ Health Perspect. 2012 Jan 9;120(4):530–4. doi: 10.1289/ehp.1104079 (PMC3339459; doi:10.1289/ehp.1104079)
Supplement: (221 KB) PDF [file ehp.1104079.s001.pdf]

Supplemental Material

An Investigation of Modifying Effects of Metallothionein Single Nucleotide Polymorphisms on the Association between Mercury Exposure and Biomarker Levels

Author and Affiliations

Yi Wang[1], Jaclyn M. Goodrich[1], Brenda Gillespie[2], Robert Werner[1,3], Niladri Basu[1], Alfred Franzblau[1*]

[1] Department of Environmental Health Sciences, School of Public Health, University of Michigan, Ann Arbor

[2] Department of Biostatistics, School of Public Health, University of Michigan, Ann Arbor

[3] Department of Physical Medicine and Rehabilitation, University of Michigan, Ann Arbor

University of Michigan School of Public Health, 1415 Washington Heights, Ann Arbor, MI USA 48109

* Corresponding author:

Alfred Franzblau, M.D.

M6023 SPH II, 1415 Washington Heights, University of Michigan School of Public Health, Ann Arbor, MI 48109-2029, USA

Email: afranz@umich.edu

Office: +1 (734)936-0758   Fax: +1 (734)763-8095

Table of Contents

Supplemental Material Figure 1. a: Scatter Plot of Ln-transformed Urine Hg against Number of Personal Amalgams Stratified by MT1M (rs2270836) Genotype; b: Scatter Plot of Ln-transformed Urine Hg against Number of Amalgam Handled Stratified by MT1M (rs2270836) Genotype; c: Scatter Plot of Ln-transformed Hair Hg against Estimated Daily Hg Intake from Fish Stratified by MT1M (rs9936741) Genotype; d: Scatter Plot of Ln-transformed Hair Hg against Estimated Daily Hg Intake from Fish Stratified by MT1A (rs8052394) Genotype; …………………………………………………………………………………………4

Supplemental Material Table 1. Summary of Average Hg Concentration in Fish Species……………………………………………………………………………….5

Supplemental Material Table 2. Arithmetic Mean Urine Hg levels (µg/L) Stratified by Levels of Elemental Hg Exposures from Occupational Practice and Personal Amalgam……………………………………………………………………………..6

Supplemental Material Table 3. Mean Hair Hg Levels (µg/g) Stratified by Levels of Estimated Methylmercury Intake from Dietary Fish Consumption…………..…7

Supplemental Material Table 4. Arithmetic Mean Urinary Hg Levels (µg/L) and Hair Hg Levels (µg/g) Stratified by Genotypes of Selected Metallothionein SNPs……………….……………………………………………………………….…..8

Supplemental Material Table 5. Coefficients and p Values from Multivariate Linear Regression Models of Natural Log-transformed Urinary Hg Predicted

against Exposure Surrogates of Elemental Hg, SNP Genotypes and Exposure-SNP Interactions……………………………………………………………….…9

Supplemental Material Table 6. Coefficients and p Values from Multivariate Linear Regression Models of Natural Log-transformed Hair Hg Predicted by Estimated Methylmercury Exposure, SNP Genotypes and Intake-SNP Interactions…………………………………………………………………….…11

Reference………………………………………………………………………….12

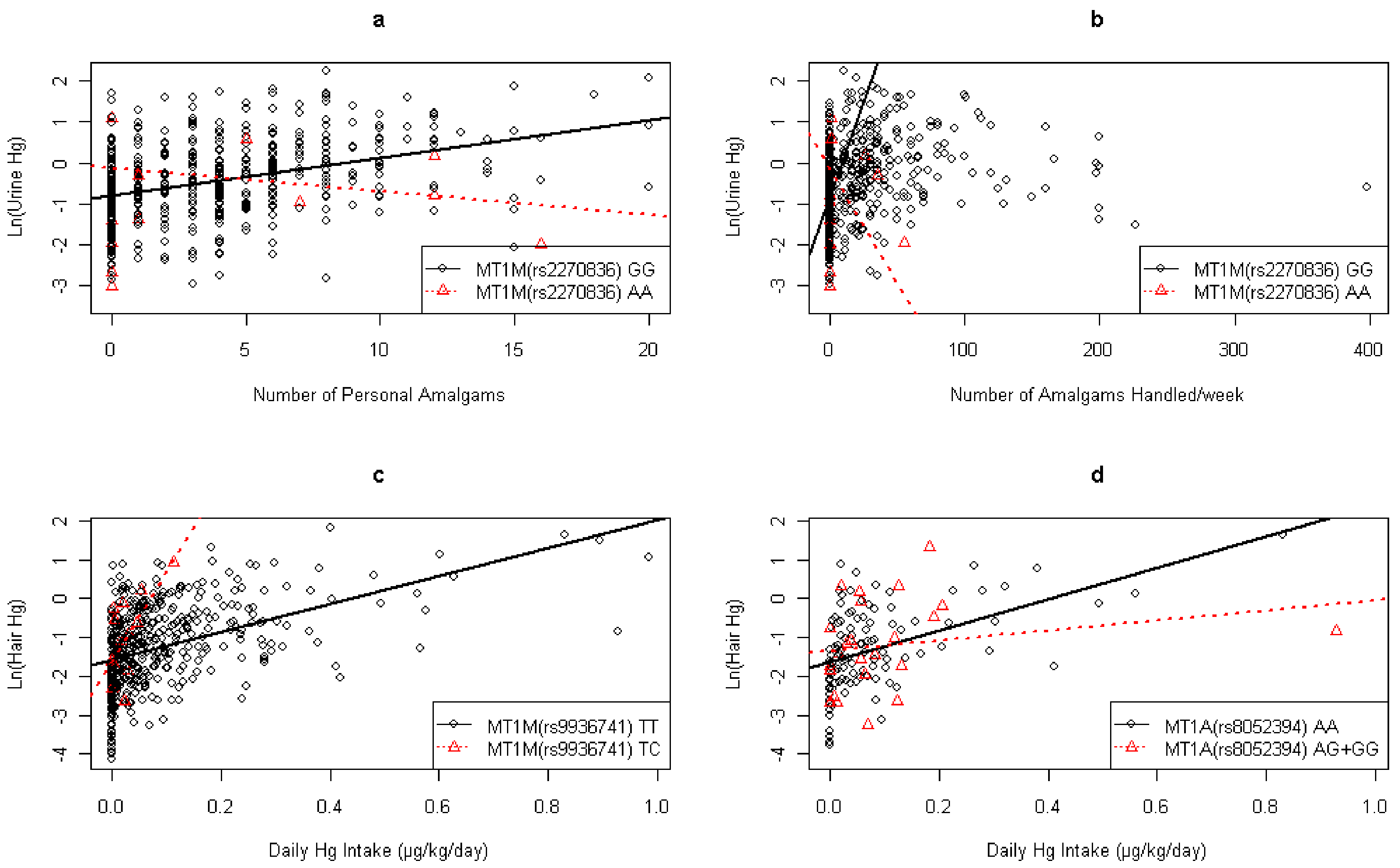

Supplemental Material, Figure 1. a: Scatter Plot of Ln-transformed Urine Hg against Number of Personal Amalgams Stratified by MT1M (rs2270836) Genotype; b: Scatter Plot of Ln-transformed Urine Hg against Number of Amalgam Handled Stratified by MT1M (rs2270836) Genotype; c: Scatter Plot of Ln-transformed Hair Hg against Estimated Daily Hg Intake from Fish Stratified by MT1M (rs9936741) Genotype; d: Scatter Plot of Ln-transformed Hair Hg against Estimated Daily Hg Intake from Fish Stratified by MT1A (rs8052394) Genotype

Supplemental Material, Table 1. Summary of Average Hg Concentrations in Fish species

Fish Species	Average Concentration (µg Hg/g)	Source	Year of Data	Number of Samples Averaged
Tuna White(canned)	0.356	FDA Monitoring Program [a]	2003	27
Tuna Light(canned)	0.258	FDA monitoring program	2003	12
Tuna Fresh	0.647	FDA monitoring program	2001-2004	9
Salmon	0.015	FDA monitoring program	2003	1
Shrimp	0.028	FDA monitoring program	1993-1995	2
Cod	0.084	FDA monitoring program	2004	19
Crab	0.049	FDA monitoring program	2004-2005	4
Scallop	0	FDA monitoring program	1991	1
Mussel	0.091	Mierzykowski et al. 2001	-	-
Halibut	0.232	FDA monitoring program	2004	14
Lobster	0.224	FDA monitoring program	2004	10
Clam	0	FDA monitoring program	1991-1993	6
Oyster	0.014	FDA monitoring program	2004	4
Perch	0.005	FDA monitoring program	1991, 1994	6
Perch Freshwater	0.141	FDA monitoring program	1991, 1995	5
Trout	0.111	FDA monitoring program	2002-2004	17
Carp	0.271	FDA monitoring program	1993	1
Walleye	0.065	FDA monitoring program	1991-1994	4
Seabass	0.679	FDA monitoring program	1996-1997	3
Fresh Seabass	0.38	Bahnick et al. 1994	-	-
Pike	0.31	Bahnick et al. 1994	-	-
Swordfish	1.312	FDA monitoring program	2002-2004	13
Red Snapper	0.193	FDA monitoring program	2002-2004	36
Shark	1.086	FDA monitoring program	1992-2000	29
King Mackerel	0.73	American Heart Association Fish 101[b]	-	-
Porgy	0.522	NOAA Technical Report 1978	-	-
Tilapia	0.01	FDA monitoring program	1991-1992	9
Whitefish	0.074	FDA monitoring program	2002-2004	19

[a] US Food and Drug Administration 1990-2010
[b] American Heart Association 20 May 2010

Supplemental Material, Table 2. Arithmetic Mean Urine Hg levels (µg/L) Stratified by Levels of Elemental Hg Exposures from Occupational Practice and Personal Amalgam

Amalgam handled/wk (N)	Urine Hg (SD)(µg/L)*	Personal amalgam (N)	Urine Hg (SD) (µg/L)*
0 (215)	0.70(0.71)	0 (128)	0.61(0.79)
1-20 (102)	1.16(1.48)	1-3 (127)	0.91(0.90)
21-45 (102)	1.36(1.32)	4-6 (132)	1.11(1.32)
>=46 (82)	1.50(1.62)	>=7 (114)	1.67(1.61)

* Cochran-Armitage trend test : $p<0.05$
SD: standard deviation

Supplemental Material, Table 3. Mean Hair Hg Levels (µg/g) Stratified by Levels of Estimated Methylmercury Intake from Dietary Fish Consumption

	Daily MeHg Intake (I) (µg/kg/day) (N)	Mean Hair Hg (SD)*
Hair Hg (µg/g)	0<=I<0.01 (145)	0.22 (0.28)
	0.01<=I<0.05 (137)	0.39 (0.39)
	0.05<=I<0.12 (112)	0.56 (0.48)
	I>=0.12 (109)	0.91 (1.02)

* Cochran-Armitage linear trend test: $p<0.05$
SD: standard deviation

Supplemental Material, Table 4: Arithmetic Mean Urinary Hg Levels (μg/L) and Hair Hg Levels (μg/g) Stratified by Genotypes of Selected Metallothionein SNPs

		Mean Urinary Hg (μg/L)					Mean Hair Hg (μg/g)				
SNP name	db SNP	N	Homozygote (N)	Heterozygote (N)	Homozygote variant (N)	ANOVA *p*	N	Homozygote (N)	Heterozygote (N)	Homozygote variant (N)	ANOVA *p*
SNPs Analyzed in Both 2009-2010											
MT2A 3'UTR(G>C)	rs10636	464	1.04(251)	1.04(171)	1.22(42)	0.65	473	0.50(254)	0.43(174)	0.57(45)	0.28
MTF1 3'UTR(G>A)	rs473279	463	1.04(215)	1.06(206)	1.17(42)	0.81	472	0.47(217)	0.51(212)	0.43(43)	0.64
MT1M 3'UTR(T>C)	rs9936741	465	1.05(449)	1.09(16)	-	0.88	474	0.48(459)	0.55(15)	-	**0.**65
MT1M 3'UTR(G>A)	rs2270836	460	1.04(329)	1.10(120)	0.98(11)	0.88	469	0.47(337)	0.51(122)	0.33(10)	0.69
MT1A missense(A>C)	rs11640851	447	1.03(190)	1.09(202)	0.97(55)	0.76	454	0.44(194)	0.51(205)	0.52(55)	0.51
SNPs Analyzed in 2009											
MTF1 3'UTR(T>C)	rs3748682	220	1.17(125)	1.05(81)	1.15(14)	0.78	221	0.48(127)	0.63(81)	0.44(13)	0.30
MT1M missense(A>C)	rs1827210	221	1.09(162)	1.22(51)	1.13(9)	0.81	223	0.50(165)	0.67(50)	0.32(8)	0.26
MT1G 3'UTR(G>T)	rs12315	222	1.11(201)	1.18(20)	0.54(1)	0.87	223	0.53(202)	0.56(20)	1.72(1)	0.26
MT1E 3'UTR(G>T)	rs708274	223	1.12(171)	1.13(49)	0.48(3)	0.67	224	0.55(174)	0.50(47)	0.34(3)	0.81
MT4 missense(G>A)	rs11643815	221	1.07(167)	1.20(51)	1.96(3)	0.39	222	0.53(166)	0.55(52)	0.33(4)	0.84
SNPs Analyzed in 2010											
MT2A 5'UTR(A>G)	rs28366003	239	1.03(213)	0.76(24)	0.34(2)	0.40	247	0.44(220)	0.39(25)	0.43(2)	0.88
MT1A missense(A>G)	rs8052394	131	0.95(109)	0.89(21)	0.28(1)	0.82	136	0.50(113)	0.44(22)	0.16(1)	0.87
MT1A 5'near gene(C>G)	rs9922957	234	1.02(180)	0.95(50)	0.69(4)	0.82	242	0.45(184)	0.37(54)	0.43(4)	0.60

[a] When number of homozygote variant is smaller than 9 and 8 for urine Hg and hair Hg, respectively, the ANOVA results shown are for comparing homozygote and the pooled heterozygote and homozygote variant.

Supplemental Material, Table 5: Coefficients and p Values from Multivariate Linear Regression Models of Natural Log-transformed Urinary Hg Predicted against Exposure Surrogates of Elemental Hg, SNP Genotypes and Exposure-SNP Interactions

		Base model							SNP main effects			
SNP name	db SNP	R^2	Personal Amalgam		Amalgam handled/week		Nondentist		Heterozygote		Homozygote variant	
			**β**	***p***	**β**	***p***	**β**	***p***	**β**	***p***	**β**	***p***
2009-2010												
MT2A 3'UTR(G>C)	rs10636	0.24	0.080	*<0.0001*	0.06	*0.37*	-0.54	*0.0002*	-0.19	*0.41*	-0.02	*0.96*
MTF1 3'UTR(G>A)	rs473279	0.24	0.086	*<0.0001*	0.18	*0.01*	-0.28	*0.06*	0.36	*0.11*	0.28	*0.43*
MT1M 3'UTR(T>C)	rs9936741	0.24	0.081	*<0.0001*	0.13	*0.01*	-0.42	*<0.0001*	0.52	*0.44*	-	-
MT1M 3'UTR(G>A)	rs2270836	0.25	0.085	*<0.0001*	0.11	*0.04*	-0.38	*0.001*	-0.04	0.87	**1.85**	***0.01***
MT1A missense(A>C)	rs11640851	0.24	0.084	*<0.0001*	0.15	*0.02*	-0.40	*0.007*	0.12	*0.60*	0.36	*0.32*
2009												
MTF1 3'UTR(T>C)	rs3748682	0.24	0.069	*<0.0001*	0.04	*0.71*	-0.69	*0.003*	-0.62	*0.10*	-0.32	*0.75*
MT1M missense(A>C)	rs1827210	0.24	0.068	*<0.0001*	0.14	*0.13*	-0.37	*0.08*	-0.33	*0.42*	1.00	*0.20*
MT1G 3'UTR(G>T)	rs12315	0.24	0.063	*<0.0001*	0.15	*0.06*	-0.41	*0.02*	-0.34	*0.54*	-	-
MT1E 3'UTR(G>T)	rs708274	0.23	0.076	*<0.0001*	0.15	*0.09*	-0.32	*0.11*	0.21	*0.61*	-	-
MT4 missense(G>A)	rs11643815	0.23	0.063	*<0.0001*	0.13	*0.14*	-0.39	*0.04*	-0.40	*0.38*	-	-
2010												
MT2A 5'UTR(A>G)	rs28366003	0.26	0.091	*<0.0001*	0.09	*0.13*	-0.54	*0.0001*	-0.43	*0.32*	-	-
MT1A missense(A>G)	rs8052394	0.23	0.127	*<0.0001*	0.06	*0.4*	-0.43	*0.02*	0.46	*0.40*	-	-
MT1A 5'near gene(C>G)	rs9922957	0.24	0.100	*<0.0001*	0.048	*0.45*	-0.48	*0.0007*	0.06	*0.87*	-	-

Supplemental Material, Table 5 (Cont.): Coefficients and p Values from Multivariate Linear Regression Models of Natural Log-transformed Urinary Hg Predicted against Exposure Surrogates of Elemental Hg, SNP Genotypes and Exposure-SNP Interactions

		**SNP-exposure interactions**											
SNP name	db SNP	Amalgam X heterozygote		Amalgam handled X heterozygote		Nondentist X heterozygote		Amalgam X homozygote variant		Amalgam handled X homozygote variant		Nondentist X homozygote variant	
		**β**	***p***	**β**	***p***	**β**	***p***	**β**	***p***	**β**	***p***	**β**	***p***
2009-2010													
MT2A 3'UTR(G>C)	rs10636	-0.005	*0.80*	0.13	*0.17*	0.23	*0.29*	0.04	*0.27*	0.007	*0.96*	-0.002	*0.99*
MTF1 3'UTR(G>A)	rs473279	-0.012	*0.55*	-0.15	*0.12*	-0.36	*0.09*	0.004	*0.91*	-0.11	*0.49*	-0.20	*0.56*
MT1M 3'UTR(T>C)	rs9936741	-0.01	*0.86*	-0.22	0.41	-0.60	0.27	-		-		-	-
MT1M 3'UTR(G>A)	rs2270836	-0.002	*0.92*	0.11	0.32	-0.03	0.90	**-0.25**	***0.02***	**-0.97**	***0.01***	-0.05	*0.94*
MT1A missense(A>C)	rs11640851	-0.003	*0.88*	-0.055	*0.58*	-0.09	*0.70*	-0.013	*0.71*	-0.12	*0.40*	-0.13	*0.69*
2009													
MTF1 3'UTR(T>C)	rs3748682	0.011	*0.73*	0.23	*0.18*	0.68	*0.06*	0.02	*0.86*	0.20	*0.60*	0.56	*0.43*
MT1M missense(A>C)	rs1827210	0.009	*0.76*	0.25	*0.20*	0.25	*0.55*	-0.01	*0.90*	-0.63	*0.15*	-0.41	*0.54*
MT1G 3'UTR(G>T)	rs12315	0.06	*0.13*	-0.20	*0.63*	-0.12	*0.84*	-	-	-	-	-	-
MT1E 3'UTR(G>T)	rs708274	-0.009	*0.78*	0.03	*0.90*	-0.24	*0.56*	-	-	-	-	-	-
MT4 missense(G>A)	rs11643815	0.04	*0.23*	0.15	*0.45*	0.16	*0.72*	-	-	-	-	-	-
2010													
MT2A 5'UTR(A>G)	rs28366003	0.03	*0.60*	-0.08	*0.63*	0.45	*0.22*	-	-	-	-	-	-
MT1A missense(A>G)	rs8052394	-0.09	*0.09*	0.04	*0.86*	-0.05	*0.92*	-	-	-	-	-	-
MT1A 5'near gene(C>G)	rs9922957	-0.03	*0.45*	0.08	*0.56*	-0.04	*0.90*	-	-	-	-	-	-

[a] When number of homozygote variant is smaller than 9, the ANOVA results shown is for comparing homozygote and the pooled heterozygote and homozygote variant.

Supplemental Material, Table 6: Coefficients and p Values from Multivariate Linear Regression Models of Natural Log-transformed Hair Hg Predicted by Estimated Methylmercury Exposure, SNP Genotypes and Intake-SNP Interactions

		Base model			SNP main effects				SNP-intake interactions			
SNP Name	db SNP	R^2	Estimated Daily MeHg Intake		Heterozygote		Homozygote variant		Intake X Heterozygote		Intake X homozygote variant	
			**β**	***p***	**β**	***p***	**β**	***p***	**β**	***p***	**β**	***p***
2009-2010												
MT2A 3'UTR(G>C)	rs10636	0.19	3.52	*<0.0001*	-0.15	*0.17*	-0.10	*0.56*	0.38	*0.65*	0.45	*0.68*
MTF1 3'UTR(G>A)	rs473279	0.19	4.52	*<0.0001*	0.13	*0.23*	-0.14	*0.50*	1.42	*0.06*	0.15	*0.93*
MT1M 3'UTR(T>C)	rs9936741	0.20	3.69	*<0.0001*	-0.03	*0.92*	-	-	**19.3**	***0.02***	-	-
MT1M 3'UTR(G>A)	rs2270836	0.19	3.93	*<0.0001*	-0.04	*0.72*	-0.14	*0.78*	-0.67	0.36	-5.33	*0.68*
MT1A missense(A>C)	rs11640851	0.18	3.74	*<0.0001*	-0.007	*0.95*	-0.09	*0.61*	-0.24	*0.78*	-0.22	*0.84*
2009												
MTF1 3'UTR(T>C)	rs3748682	0.24	4.97	*<0.0001*	0.32	*0.06*	0.21	*0.57*	-1.63	*0.13*	1.23	*0.73*
MT1M missense(A>C)	rs1827210	0.23	4.4	*<0.0001*	0.05	*0.81*	-0.05	*0.93*	-0.61	*0.56*	-1.36	*0.98*
MT1G 3'UTR(G>T)	rs12315	0.23	4.21	*<0.0001*	0.30	*0.38*	-	-	-1.45	*0.52*	-	-
MT1E 3'UTR(G>T)	rs708274	0.24	4.79	*<0.0001*	-0.05	*0.77*	-	-	-1.57	*0.13*	-	-
MT4 missense(G>A)	rs11643815	0.24	3.97	*<0.0001*	-0.23	*0.21*	-	-	0.73	*0.54*	-	-
2010												
MT2A 5'UTR(A>G)	rs28366003	0.14	3.08	*<0.0001*	-0.17	*0.51*	-	-	0.97	*0.60*	-	-
MT1A missense(A>G)	rs8052394	0.21	4.04	*<0.0001*	0.21	*0.40*	-	-	**-300**	***0.02***	-	-
MT1A 5'near gene(C>G)	rs9922957	0.15	3.52	*<0.0001*	-0.22	*0.19*	-	-	-1.04	*0.36*	-	-

Reference

American Heart Association. 20 May 2010. Fish 101. Available: http://www.heart.org/HEARTORG/GettingHealthy/NutritionCenter/Fish-101_UCM_305986_Article.jsp#fish_with_high_levels_of_mercury [accessed 11/04/2010]

Bahnick D, Sauer C, Butterworth B, Kuehl D. 1994. A national study of mercury contamination of fish. Chemosphere 29:537–546.

Mierzykowski S.E. and K.C. Carr. 2001. Total mercury and methylmercury in freshwater mussels *Elliptio complanata* from the Sudbury River watershed, Massachusetts. USFWS. Special Project Report FY98-MEFO-2-EC. Maine Field Office. Old Town, ME.

Manooch, Charles S., III, and William W. Hassler. 1978. Synopsis of biological data on the red porgy, *Pagrus pagrus* (Linnaeus). NOAA Technical Report NMFS Circular 412:9.

US Food and Drug Administration.1990-2010. FDA monitoring program (1990-2010). Available: http://www.fda.gov/Food/FoodSafety/Product-SpecificInformation/Seafood/FoodbornePathogensContaminants/Methylmercury/ucm191007.htm [accessed 11/04/2010]
